# Antithrombotic Therapy for Percutaneous Cardiovascular Interventions: From Coronary Artery Disease to Structural Heart Interventions

**DOI:** 10.3390/jcm8112016

**Published:** 2019-11-19

**Authors:** Alessandro Caracciolo, Paolo Mazzone, Giulia Laterra, Victoria Garcia-Ruiz, Alberto Polimeni, Salvatore Galasso, Francesco Saporito, Scipione Carerj, Fabrizio D’Ascenzo, Guillaume Marquis-Gravel, Gennaro Giustino, Francesco Costa

**Affiliations:** 1 Department of Clinical and Experimental Medicine, Policlinic “G. Martino”, University of Messina, 98100 Messina, Italy; caracciolo.alessandro.ac@gmail.com (A.C.); mazzonepaolo89@gmail.com (P.M.); giulia.laterra1990@gmail.com (G.L.); sgalasso@gmail.com (S.G.); fsaporito@unime.it (F.S.); scarerj@unime.it (S.C.); 2 UGC del Corazón, Servicio de Cardiología, Hospital Clínico Universitario Virgen de la Victoria, 29010 Málaga, Spain; mavigaru@gmail.com; 3 Division of Cardiology, Department of Medical and Surgical Sciences, Magna Graecia University, 88100 Catanzaro, Italy; polimeni@unicz.it; 4 Division of Cardiology, Department of Medical Sciences, Città della Salute e della Scienza, University of Turin, 10124 Turin, Italy; fabrizio.dascenzo@gmail.com; 5 Duke Clinical Research Institute, Durham, NC 27708, USA; guillaume.marquis.gravel@duke.edu; 6 Montreal Heart Institute, Montreal, QC H1T 1C8, Canada; 7 The Zena and Michael A. Wiener Cardiovascular Institute, Icahn School of Medicine at Mount Sinai, New York, NY 10029-6574, USA; gennaro.giustino@mountsinai.org; 8 Department of Population Health Science and Policy, Icahn School of Medicine at Mount Sinai, New York, NY 10029-6574, USA

**Keywords:** dual antiplatelet therapy, antiplatelet, coronary artery disease, structural heart disease

## Abstract

Percutaneous cardiovascular interventions have changed dramatically in recent years, and the impetus given by the rapid implementation of novel techniques and devices have been mirrored by a refinement of antithrombotic strategies for secondary prevention, which have been supported by a significant burden of evidence from clinical studies. In the current manuscript, we aim to provide a comprehensive, yet pragmatic, revision of the current available evidence regarding antithrombotic strategies in the domain of percutaneous cardiovascular interventions. We revise the evidence regarding antithrombotic therapy for secondary prevention in coronary artery disease and stent implantation, the complex interrelation between antiplatelet and anticoagulant therapy in patients undergoing percutaneous coronary intervention with concomitant atrial fibrillation, and finally focus on the novel developments in the secondary prevention after structural heart disease intervention. A special focus on treatment individualization is included to emphasize risk and benefits of each therapeutic strategy.

## 1. Introduction

The link between percutaneous cardiovascular intervention and antithrombotic therapy begins right at the time of the first coronary angioplasties performed by Andreas Grüntzig. After the introduction of this technique in 1977, post-procedural warfarin therapy was initiated for 6–9 months after intervention to reduce the risk of post-procedural ischemic complications [1]. A few years later, the same group tested whether aspirin (ASA), with its secondary inhibition capacity of platelet’s thromboxane A2 pathway, was superior to the oral anticoagulation in decreasing the incidence of reintervention after PCI [2]. While the techniques and the devices were improved in the following years, the introduction of the coronary stent and its first-in-man implantation in 1986 by Jacques Puel, dramatically improved early and late results of percutaneous coronary intervention (PCI), yet introducing a novel clinical issue: stent thrombosis. This complication occurred in up to 10% of cases at that time, and while a treatment with warfarin alone or warfarin plus aspirin was common after PCI and stent implantation, the rate of stent thrombosis remained high. Only in 1998 two randomized trials demonstrated that dual antiplatelet therapy (DAPT), constituted by the combination of ASA and a platelet P2Y12 inhibitor, was more effective than warfarin and aspirin therapy in reducing the risk of stent thrombosis [3,4]. These studies have established DAPT the as the gold standard therapy after angioplasty and stent implantation [5,6,7]. At the same time, similar antithrombotic practices were implemented for other percutaneous cardiovascular interventions such as atrial septal defect [8,9] or patent foramen ovale (PFO) closure [5,10], and more recently transcatheter aortic valve implantation (TAVI) [11] and transcatheter mitral valve repair (TMVR), in which the evidence coming from the coronary intervention field has been extrapolated to structural heart interventions. The aim of the current review is to summarize the evidence that established the current standards and recommendation for DAPT type and duration after coronary intervention, comment on the current standards for personalized treatment decision-making, and discuss the most recent advances in terms of treatment combination of antiplatelet and direct acting oral anticoagulants (DOAC) for the secondary prevention of high ischemic risk patients and patients with concomitant PCI and non-valvular atrial fibrillation. Finally, we touch upon the current evidence, recommendations and future perspectives for antithrombotic therapy in patients undergoing structural heart interventions.

## 2. Type of Antithrombotic Treatment after PCI

### 2.1. Evidence for Clopidogrel

While ticlopidine was the first P2Y12 inhibitor to be associated with ASA for DAPT, its worse safety profile made it obsolete after the introduction of clopidogrel. In 2000, the CLASSIC study was the first comparative trial in which clopidogrel in association with ASA was compared with ticlopidine in association with ASA after PCI [12]. In this study, which randomized 1020 patients to the combination of clopidogrel and ASA vs. ticlopidine and ASA, clopidogrel provided better safety and tolerability (i.e., less allergy, skin or gastrointestinal disorders and neutropenia) with a similar bleeding and efficacy profile. The CURE trial compared clopidogrel vs. placebo in addition to ASA in patients who presented with acute coronary syndromes without ST-segment elevation undergoing invasive or non-invasive management. The first primary outcome—a composite of death from cardiovascular causes, nonfatal myocardial infarction, or stroke—was significantly reduced in the clopidogrel group by 20% as compared to placebo (relative risk (RR) 0.80; 95% CI 0.72–0.90; *p* < 0.001) [13]. The evidence provided by the landmark CURE trial established DAPT with clopidogrel as the standard of care after acute coronary syndrome (ACS) and after coronary stent implantation. However, its high inter-individual variability in platelet inhibition and a sizable proportion of non-responders patient [14] highlighted the need for more potent and consistent platelet inhibition that would be introduced with novel generation P2Y12 inhibitors. 

### 2.2. Evidence for Prasugrel

**Figure 1:**
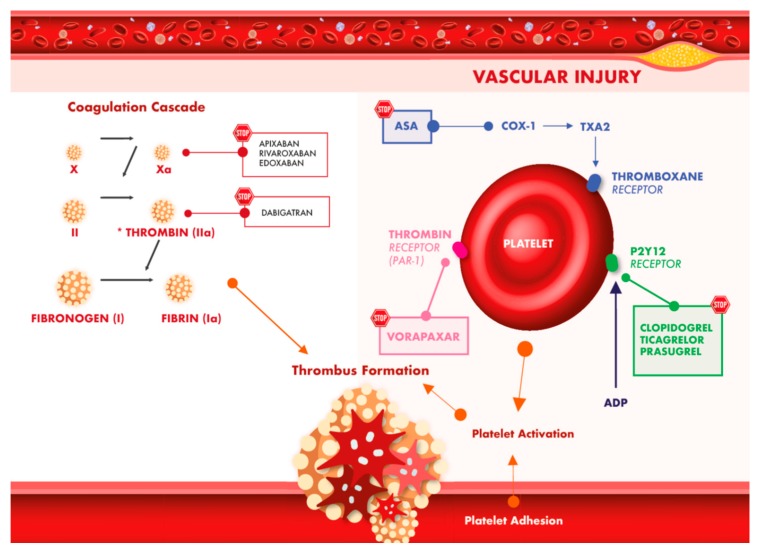
Antiplatelet and anticoagulant treatment strategies for secondary prevention of ischemic events.

Similar to clopidogrel, prasugrel is a prodrug which requires conversion to an active metabolite to ultimately irreversibly bind to the P2Y12 receptor and achieve antiplatelet effect (Figure 1). different from clopidogrel, prasugrel has a more rapid and greater antiplatelet effect [15,16]. These drugs have been tested head-to-head in TRITON TIMI 38 trial. In this study, 13,608 patients with moderate to high-risk ACS and a scheduled invasive strategy were randomized to receive either prasugrel or clopidogrel. The primary efficacy endpoint of death from cardiovascular causes, nonfatal myocardial infarction, or nonfatal stroke occurred in 12.1% of patients receiving clopidogrel and 9.9% of patients receiving prasugrel (HR 0.81; 95% confidence interval (CI)), 0.73–0.90; *p* < 0.001). There was also a significant reduction of myocardial infarction (9.7% for clopidogrel vs. 7.4% for prasugrel; *p* < 0.001), urgent target-vessel revascularization (3.7% vs. 2.5%; *p* < 0.001), and stent thrombosis (2.4% vs. 1.1%; *p* < 0.001) related to the use of Prasugrel. However, the higher efficacy of prasugrel was counterbalanced by an increased risk of major bleeding, including fatal bleeding. Patients with previous stroke or transient ischemic attack were harmed by prasugrel use (hazard ratio, 1.54; 95% CI, 1.02–2.32; *p* = 0.04), while patients with 75 years of age or older and patients weighing less than 60 kg had had no benefit from prasugrel compared to ticagrelor [17]. TRITON TIMI 38 did not include patients with ACS undergoing medical-management. This population was specifically evaluated in the TRILOGY ACS trial. In TRILOGY ACS, patients were randomly allocated to prasugrel or clopidogrel and managed exclusively with medical therapy without revascularization. Prasugrel ultimately failed to show superiority for the primary study outcome compared to Clopidogrel in ACS patient treated medically without revascularization [18]. 

Finally, the recent SASSICAIA trial (NCT02548611) explored the impact of prasugrel in patients undergoing elective PCI. In this study, patients with stable CAD and undergoing PCI were randomized to a loading dose with prasugrel or clopidogrel at the time of PCI. All patients after PCI were treated with clopidogrel alone and the primary outcome of all-cause death, any myocardial infarction, definite/probable stent thrombosis, stroke, or urgent vessel revascularization at 30 days was evaluated. Ultimately, there was no difference for the primary endpoint in the two study arms; hence, SASSICAIA trial failed to demonstrate that a prasugrel loading dose in elective patients reduces ischemic events as compared to an initial loading dose of clopidogrel. 

### 2.3. Evidence for Ticagrelor

Ticagrelor is a P2Y12 inhibitor which binds reversibly the platelet receptor with a shorter plasma half-life. This has a more rapid onset and more pronounced platelet inhibition compared to clopidogrel (Figure 1) [19]. In the PLATO trial, 18,624 patients with acute coronary syndrome were randomized to Ticagrelor or Clopidogrel. In this study ticagrelor significantly reduced the rate of death from vascular causes, myocardial infarction, or stroke (9.8% vs. 11.7%; HR 0.84; 95% CI, 0.77–0.92; *p* < 0.001), but also increased non-coronary artery by-pass graft (CABG) related major bleeding [20]. In the ST Elevation Myocardial Infarction to Open the Coronary Artery (ATLANTIC) [21] study, 1862 patients with STEMI were randomized to pre-hospital administration of the loading dose of ticagrelor administered directly in the ambulance vs. in-hospital administration in the catheterization laboratory [21]. The two co-primary endpoints explored in the study (>70% resolution of ST-segment elevation before PCI and proportion of TIMI flow grade 3 at initial angiography) did not differ significantly between the two groups. The rates of definite stent thrombosis were lower in the pre-hospital administration group than in the in-hospital group (0% vs. 0.8%, *p* = 0.008 in the first 24 h; 0.2% vs. 1.2%, *p* = 0.02 at 30 days). No difference for major bleeding was observed with the two strategies.

### 2.4. Evidence for the Direct Comparison of Prasugrel and Ticagrelor

Despite having different pharmacological characteristics, prasugrel and ticagrelor demonstrated a similar efficacy profile in terms of platelet inhibition and percentage of patients with high on-treatment platelet reactivity [22]. On a clinical standpoint, while the two P2Y12 inhibitors proved superiority compared to clopidogrel in the two registration studies [17,20], a direct comparison between the two drugs has been provided only recently [23]. In the PRAGUE-18 study, 1230 patients with acute myocardial infarction (MI) treated with primary PCI were randomized to prasugrel or ticagrelor. The intended treatment duration was 12 months [24]. At one-year follow-up (although the study was ended prematurely for futility), the combined endpoint of cardiovascular death, MI, or stroke occurred in 6.6% of prasugrel and in 5.7% of ticagrelor patients (HR: 1.167; 95% CI: 0.742–1.835; *p* = 0.503). No significant differences for all bleeding (10.9% vs. 11.1%; *p* = 0.999) and TIMI major bleeding (0.9% vs. 0.7%; *p* = 0.754) were observed [24]. Rafique et al. explored the direct comparison of two P2Y12 inibhitors in the setting of primary PCI executing a network metanalysis of 37 studies: at one-year follow-up, prasugrel compared to ticagrelor was associated with a lower rate of MACE (OR: 0.77, 95% CI: 0.61–0.97), death for all causes (OR: 0.63, 95% CI: 0.46–0.87), but not CV death, MI or ST, with a similar rate of major bleeding [25]. While prior studies and meta-analysis have been inconclusive in unraveling possible differences in clinical outcomes between the two treatments, ISAR-REACT 5 was the first trial properly designed and sized to evaluate the direct comparison of ticagrelor vs. prasugrel [26]. In this multicenter, investigator-initiated, open-label trial 4018 patients with ACS undergoing invasive management were randomly assigned to either ticagrelor or prasugrel at the standard loading and maintenance doses. Importantly, according to guideline recommendations, patients randomized to the prasugrel arm were loaded only after angiography and to be assigned to a reduced maintenance dose if body weight was <60 kg or age >75 years. The primary endpoint of the study was a composite of death, myocardial infarction, or stroke at one year. The major secondary endpoint was bleeding according to BARC 3, 4 or 5 definition. At 12-month follow-up, the primary endpoint occurred 9.3% in the ticagrelor group and in 6.9% in the prasugrel group (HR, 1.36; 95% confidence interval (CI), 1.09–1.70; *p* = 0.006). The individual elements of the primary endpoint were all numerically in favor of prasugrel (death, 4.5% vs. 3.7%; MI, 4.8% vs. 3.0%; stroke, 1.1% vs. 1.0%). Stent thrombosis was similar between the two treatments. Surprisingly, no difference in terms of bleeding was observed among the two treatment arms, with a rate of BARC 3, 4 or 5 of 5.4% in the ticagrelor arm and 4.8% the prasugrel arm (HR 1.12; 95% CI, 0.83–1.51; *p* = 0.46) [26]. 

## 3. Duration of DAPT after PCI and Major Determinants for Treatment Selection

### Randomized Trials

Numerous studies have tried in recent years to clarify which temporal window could be ideal after PCI or ACS (Table 1), and in general two major strategies have been presented relative to what was considered the standard of care for DAPT duration of 12 months [27]. A short DAPT encompasses a strategy of less than 12 months of treatment, and a long DAPT extends its duration beyond 12 months. With respect to a strategy of shortening DAPT duration, the first randomized study testing this assumption was the Efficacy of Xience/Promus Versus Cypher to Reduce Late Loss After Stenting (EXCELLENT). In this study, 1443 patients treated with drug-eluting stent (DES) implantation and randomized to 6 vs. 12 months DAPT, were evaluated to test the non-inferiority of the short DAPT strategy compared to the standard of care for the primary endpoint of cardiac death, myocardial infraction (MI), or ischemia-driven target vessel revascularization. Ultimately, six-month DAPT demonstrated non-inferior compared to the standard of care. In addition, TIMI major and minor bleeding were numerically higher in the 12-month group, but this difference was not statistically significant (HR 0.40; 95% CI: 0.13–1.27; *p* = 0,12) [28]. A similar hypothesis of non-inferiority of 6 vs. 12 months DAPT was also tested and observed in The Second Generation Drug-Eluting Stent Implantation Followed by Six-Versus Twelve-Month Dual Antiplatelet Therapy (SECURITY) [29] and in the Intracoronary Stenting and Antithrombotic Regimen: Safety and Efficacy of Six-month Dual Antiplatelet Therapy After Drug-Eluting Stenting (ISAR-SAFE) [30]. A different design was implemented in the Impact of Intravascular Ultrasound Guidance on Outcomes of XIENCE PRIME Stents in Long Lesions study [31]. In this case a factorial design was implemented and the first randomization was based on the routinely use of intravascular ultrasound PCI guidance, while the second randomization was for DAPT duration (6 vs. 12 months). At 12-month follow-up the composite of Cardiac death, MI, stroke, and TIMI major bleeding was similar between patients treated with 6- or 12-month DAPT (2.2% vs. 2.1%; *p* = 0.85). Interestingly, in the subgroup analysis for the primary endpoint patients treated with intravascular ultrasound guided stent implantation benefitted more from a shorter DAPT treatment as compared to those treated with angiographic guidance alone. In the specific setting of ACS, two randomized trials tested the feasibility of a shorter DAPT duration of six months as compared to the standard of care 12 months strategy: the SMART-DATE trial randomized 2712 patients to a DAPT for 6 or 12 months. At 18 months, the primary endpoint, a composite of all-cause death, MI, or stroke was recorded equally in the two study arms (4.7% vs. 4.2%) meeting the pre-specified non-inferiority hypothesis. However, a significant excess of MI was recorded in the short DAPT cohort [32]. A more specific setting was explored in the DAPT-STEMI trial where 870 patients with STEMI treated with primary PCI and second-generation DES that after six months of treatment with DAPT were randomized to another 12 months of DAPT therapy or to stop P2Y12 inhibitor and continue with aspirin only. The primary study endpoint was a composite of death, MI, revascularization, stroke, and major bleeding at 24 months after primary PCI. Short DAPT was found to be non-inferior as compared to the standard 12-month treatment duration (short DAPT 4.8% vs. long DAPT 6.6%; *p* = 0.004). However, these results should be interpreted with caution as the study enrolled a small sample size, and the event rate recorded was low compared to other studies in the literature [33]. The short DAPT therapy in high ischemic risk patient was the goal of the REDUCE trial (NCT02118870), which selected a population with a higher baseline ischemic risk to explore the non-inferiority of 3 vs. 12 months of DAPT in patients with ACS treated with PCI. The 1496 patients included in the study have been treated at index procedure exclusively with a bioabsorbable polymer DES. The primary endpoint was a composite of all cause death, MI, stent thrombosis, stroke, target vessel revascularization, or bleeding, with a wide, 5%, non-inferiority margin. The study reached non-inferiority with an event rate of 8.2% in the short DAPT arm and 8.4% in the long DAPT arm (*p* < 0.001). However, among secondary ischemic endpoints, stent thrombosis (1.2% vs. 0.4%; *p* = 0.08) and all-cause mortality (1.9% vs. 0.8%; *p* = 0.07) were numerically higher in the short DAPT arm, a trend which was consistent with that observed in the SMART-DATE trial [32]. Different temporal strategies have been considered in the design of the studies ITALIC [34] and NIPPON [35], respectively, 6 vs. 24 months of DAPT and 6 vs. 18 months of DAPT. These trials reached non-inferiority, but, again, the results from these studies should be interpreted with caution due to the study early termination and the wide non-inferiority margin selected. 

The rationale for a long DAPT strategy derives from the residual thrombotic risk during secondary prevention, which remains high despite potent P2Y12 inhibition [36]. Maintaining on a longer term a more potent and consistent platelet inhibition with DAPT should reduce the rate of ischemic complication due to plaque progression and rupture, yet may increase the risk of bleeding complications that have an equal negative impact on mortality [37,38]. The Dual-Antiplatelet Treatment Beyond one Year After Drug-Eluting Stent Implantation (ARCTIC INTERRUPTION) trial [39], an extended follow-up of the ARCTIC study, tested the superiority of ≥18 months DAPT vs. 12 months after stent implantation after an elective PCI. The primary efficacy endpoint occurred in 4% of patients in both study arms, while a significant excess of bleeding was detected in the prolonged DAPT arm. The PROlonging Dual antIplatelet treatment after Grading stent-induced intimal hYperplasia study (PRODIGY) trial [40], randomly allocated patients to a long DAPT strategy for 24 months vs. a short DAPT for six months showing no difference between the two strategies with respect to the study primary efficacy endpoint of death, MI, stroke, whereas an excess of actionable BARC 2, 3 or 5 bleeding was noted among patients allocated to the 24 month DAPT arm. The Dual Antiplatelet Therapy (DAPT) study was the first study with the adequate power to detect a difference for more rare ischemic events including stent thrombosis [41]. The study was funded by the American Food and Drug Administration and randomized 9961 patients who tolerated an uneventful course of DAPT of 12 months after stenting to two treatment strategies: interrupting DAPT at 12 months or continuing treatment up to 30 months. Extended DAPT resulted in a 1% absolute reduction in very late stent thrombosis and a 1.6% absolute reduction of major adverse cardiovascular and cerebrovascular events (MACCE). A total reduction of 2% of MI was also noted and this was ascribed in half of the cases to a different vessel from the one treated on a first place. Despite the sound reduction of ischemic events with a prolonged DAPT, this strategy was also burdened by a significant excess of major bleeding, with a doubtful or even negative impact on all cause mortality [41,42]. A landmark trial that further supported the potential extension of DAPT to the long term is the Prevention of Cardiovascular Events in Patients with Prior Heart Attack Using Ticagrelor Compared to Placebo on a Background of Aspirin–Thrombolysis in Myocardial Infarction 54 (PEGASUS-TIMI 54) study. PEGASUS investigated the potential benefit of extended DAPT therapy in patients with a prior myocardial infarction implementing two different Ticagrelor doses (90 mg twice daily and 60 mg twice a daily) on top of aspirin compared to placebo plus aspirin. The primary outcome was cardiovascular death, MI, or stroke, which occurred in 7.8% of the ticagrelor 90 mg bid group (hazard ratio (HR) vs. placebo 0.85, *p* = 0.008), 7.8% of the ticagrelor 60 mg bid group (HR vs. placebo 0.84, *p* = 0.004), and 9.0% of the placebo group. Ticagrelor was also associated with a significant increase in TIMI major bleeding, but no increase in intracranial hemorrhage [43]. Similar results were also observed, in a different population in THEMIS-PCI trial. This study evaluated the impact of DAPT with ticagrelor plus aspirin in patients with stable ischemic heart disease, type 2 diabetes, and prior PCI, compared to aspirin alone. At a median follow-up of 39.9 months, ticagrelor on top of aspirin was associated to a reduction of the primary efficacy outcome of cardiovascular death, MI, or stroke, which occurred in 7.3% of the ticagrelor/aspirin group and in 8.6% of the placebo/aspirin group (*p* = 0.013). The higher treatment efficacy was counterbalanced by an excess of bleeding, in fact the primary safety outcome of the study, TIMI major bleeding, occurred in 2.0% of the ticagrelor/aspirin group compared to 1.1% of the placebo/aspirin group (*p* < 0.0001). No difference in intracranial hemorrhage was observed. When the exploratory composite outcome of so-called “net irreversible harm”—a composite of all-cause death, MI, stroke, fatal bleeding, or intracranial hemorrhage—was explored, this occurred in 9.3% of the ticagrelor/aspirin group and in 11.0% of the placebo/aspirin group (*p* = 0.005) [44].

While DAPT type and duration selection is clearly associated with a trade-off between ischemia and bleeding [36,45], the optimal strategy, maximizing efficacy and safety should be individualized based on the patients’ characteristics [46,47,48,49,50]. The type of clinical presentation (i.e., SCAD, unstable angina, non-STEMI, or STEMI) at the time of PCI is a major determinant of a patient’s mortality risk, and may profoundly impact the probability of ischemic recurrences [51,52,53]. In the DAPT trial, 30.7% presented with an acute coronary syndrome at the time of PCI. Long DAPT compared with standard DAPT regimen significantly reduced definite or probable stent thrombosis in patients both with MI (0.5 vs. 1.9%; *p* = 0.001) and without MI (0.4 vs. 1.1%; *p* = 0.001), yet the magnitude of the reduction of MACCE from longer DAPT was bigger among patients with MI (3.9 vs. 6.8%; *p* = 0.001) than among those without MI (4.4 vs. 5.3%; *p* = 0.08) at the time of presentation (P_int_ = 0.03) [41,54]. The ischemic risk after PCI is also associated to several anatomical or procedural characteristic [55]. The concept of PCI complexity is quantifiable using previously validated and guideline-endorsed criteria: PCI with ≥3 stents implanted and 3 ≥ lesions and/or coronary vessels treated; and/or bifurcation with 2 stents implanted, total stent length >60 mm, and/or treatment of a chronic total occlusion [56]. In these patients, long-term DAPT (≥12 month) compared with a short period of DAPT (three or six months), significantly reduced the risk of cardiac ischemic events with a greater effect in patients undergoing PCI with more complex features. On the other hand, the risk of bleeding, estimated through risk scores, is also a potent driver for treatment selection [57,58,59,60]. The PRECISE-DAPT score based on five-items (age, creatinine clearance, hemoglobin, white blood cell count, and prior spontaneous bleeding) showed potential to guide DAPT duration decision-making: individuals deemed at high bleeding risk (PRECISE-DAPT score ≥ 25), prolonged DAPT was associated with no ischemic benefit but a significant bleeding hazard, whereas, on the other side, among patients not deemed at high bleeding risk (PRECISE-DAPT score < 25) a long DAPT treatment was associated with a significant reduction in the composite ischemic endpoint of MI, definite stent thrombosis, stroke, and target vessel revascularization without a significant increase in bleeding events [58]. It is interesting to note that according to a recent retrospective study, patients who underwent complex PCI would benefit long-term DAPT only if not at high risk of bleeding at baseline (PRECISE DAPT < 25) [61,62]. Ultimately, international guidelines agree on the fact that coronary stent type is no more a driver mandating different DAPT duration, and preferential BMS use for anatomical [63] or clinical [64,65,66,67] reasons mandating shorter DAPT duration are no longer recommended [48]. 

## 4. From DAPT to Single Antiplatelet Therapy (SAPT), Evidence for Aspirin Withdrawal and Monotherapy with P2Y12 Inhibitor

**Figure 2:**
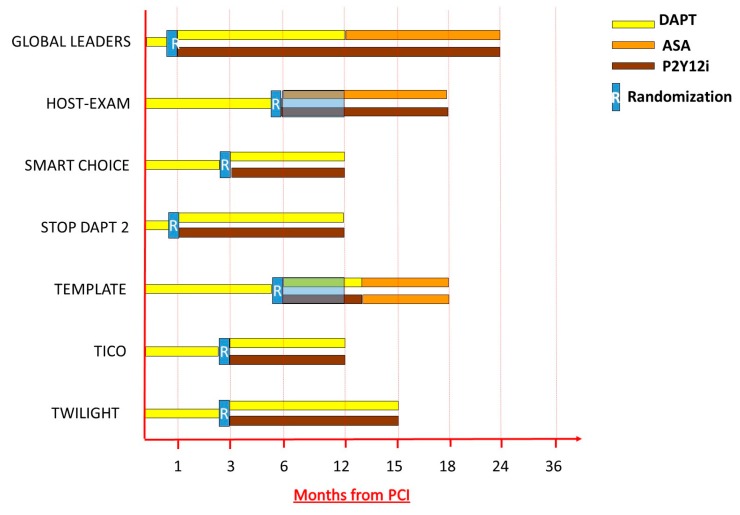
Design of clinical trials exploring aspirin withdrawal after percutaneous coronary intervention.

Several clinical studies have been designed to test efficacy and safety of a strategy of early withdrawal of aspirin after PCI (Figure 2). In the One-Month Dual Antiplatelet Therapy Followed by Clopidogrel Monotherapy vs. Standard 12-Month Dual Antiplatelet Therapy With Clopidogrel After Drug-Eluting Stent Implantation (STOPDAPT-2) study, 3045 patients undergoing PCI (62% SCAD) were randomized to one month of DAPT followed by clopidogrel monotherapy for five years versus 12 months of DAPT followed by Aspirin monotherapy for five years. After the first year of follow-up, the primary endpoint of cardiovascular death, myocardial infarction (MI), definite stent thrombosis, stroke, or TIMI major/minor bleeding occurred in 2.4% of the one-month DAPT group and in 3.7% of the 12-month DAPT group (HR 0.64; 95% CI 0.42–0.98), reaching both non-inferiority and superiority of the experimental treatment compared to the 12 month DAPT arm (*p* non-inferiority < 0.001; *p* superiority = 0.04). This result was mostly driven by a significant 74% reduction of TIMI major or minor bleeding among patients treated with shorter DAPT (HR 0.26 95% CI 0.11–0.64; *p* = 0.002) [68]. However, since this study was exclusively conducted in an Asian population and with specific procedural standards (e.g., imaging guided PCI was carried out in >97% of patients) the external validity of these results may be limited. 

Similarly, the Comparison Between P2Y12 Antagonist Monotherapy and Dual Antiplatelet Therapy After DES (SMART-CHOICE) study compared a short treatment arm of three months of DAPT followed by P2Y12 inhibitor monotherapy with the standard 12-month duration of DAPT. The primary study hypothesis was the non-inferiority of the experimental treatment as compared to the standard treatment for the primary ischemic endpoint. In total, 2993 patients undergoing PCI (58% with ACS at presentation) were ultimately included. At 12-month follow-up, the primary outcome of all-cause death, MI, or stroke occurred in 2.9% of patients in the three-month DAPT arm and in 2.5% in the 12-month DAPT arm, meeting the pre-specified non-inferiority margin (*p* for non-inferiority = 0.007; *p* for superiority = 0.46). Actionable bleeding events accounted according to bleeding academic research consortium (BARC) Type 2, 3 or 5 criteria were significantly less in the short DAPT arm, mostly due to a reduction of minor bleeding [32].

The GLOBAL LEADER trial included 15,968 patients who underwent PCI for stable or unstable coronary disease that were randomized to DAPT of aspirin plus ticagrelor for one month, followed by ticagrelor monotherapy for 23 months versus DAPT for 12 months (aspirin/clopidogrel for stable coronary disease or aspirin/ticagrelor for unstable coronary disease), followed by aspirin monotherapy for 12 months [69]. The primary outcome was a composite of all-cause death or nonfatal myocardial infarction). The primary study hypothesis was to demonstrate the superiority of the experimental treatment for the composite ischemic endpoint at 24-month follow-up. Ultimately, at 24-month follow-up, there was an equipoise for the two treatment strategies for the primary study endpoint (*p* = 0.073). Importantly, adherence to the assigned treatment was as low as 78% at 24 months in the experimental arm, which may have reduced the pre-specified statistical power of the study [69,70].

Finally, the most recent TWILIGHT trial tested the impact of aspirin withdrawal at three months after PCI in 7119 high-risk patients who were randomized at three months after PCI to ticagrelor monotherapy for additional nine months or continuing standard DAPT with aspirin and ticagrelor. The trial specifically included patients that were deemed both at high ischemic and bleeding risk. At 12 months after PCI, the primary safety endpoint, a composite of BARC 2, 3 or 5 bleeding was reduced by 44% in the ticagrelor monotherapy arm (4.0% vs. 7.1%; HR 0.56; 95% CI 0.45–0.68; *p* < 0.001). No significant differences in MACE were observed in the two study arms, consistently to what observed in other studies of aspirin withdrawal. 

Taken together, all the studies exploring aspirin withdrawal presented to date, show a consistent reduction of bleeding events by removing aspirin at different timepoints after PCI, with no apparent trade-off in ischemic events. However, some consideration regarding these studies should be highlighted. First, the STOP-DAPT 2 trial and SMART-CHOICE were exclusively conducted in an Asian population with very high usage of intravascular imaging for optimizing stent implantation, potentially limiting external validity of these findings to other centers with different practice. Second, the timing to randomization and effective aspirin withdrawal was of one month in three trials and three months in another. In addition, the baseline ischemic risk of the populations explored was different among trials; hence, it is not clear which is the optimal timing for safe aspirin withdrawal. In addition, whether potent P2Y12 inhibition should be preferred over a treatment with clopidogrel, in which high prevalence of drug non-responders may pose concerns for aspirin withdrawal should be clarified. In light of the recent results of the ISAR-REACT 5 trial, whether monotherapy with ticagrelor or with prasugrel should be preferred has not yet been studied. 

## 5. Combination of Oral Anticoagulants and Antiplatelet Therapy in Patients at Higher Ischemic Risk or with Non-Valvular Atrial Fibrillation

### 5.1. Direct-Acting Oral Anticoagulants (DOAC) in Association to Antiplatelet Therapy in Patients at High Ischemic Risk

Despite the great progress in reducing the rate of events in patients at high ischemic risk, there is still a significant residual thrombotic risk in patients with coronary artery disease and prior vascular events, which is as high as 10% at 12 months after the occurrence of an ACS. [71] Novel pharmacological strategies focused on the long-term association of antiplatelet and anticoagulant drugs to further reduce the ischemic burden have been tested (Table 2). The Anti-Xa Therapy to Lower Cardiovascular Events in Addition to Standard Therapy in Subjects with Acute Coronary Syndrome–Thrombolysis in Myocardial Infarction 51 (ATLAS ACS 2-TIMI 51) study was based on this assumption and was the first testing the hypothesis that additional factor Xa inhibition on top of standard DAPT could further reduce the residual ischemic risk of patients after an acute coronary syndrome. The ATLAS ACS 2-TIMI 51 included a total of 15,526 patients with a recent ACS to receive twice-daily doses of either 2.5 mg or 5 mg of Rivaroxaban or placebo on top of standard DAPT with Aspirin and Clopidogrel. The primary efficacy end point was a composite of death from cardiovascular causes, myocardial infarction, or stroke, while the safety outcome was TIMI major bleeding not related to coronary-artery bypass grafting (CABG). After a mean treatment of 13 months, rivaroxaban significantly reduced the primary end point, as compared with placebo (HR in the rivaroxaban group, 0.84; 95% confidence interval, 0.74–0.96; *p* = 0.008), with a significant improvement observed for both the twice-daily 2.5-mg dose (9.1% vs. 10.7%, *p* = 0.02) and the twice-daily 5-mg dose (8.8% vs. 10.7%, *p* = 0.03). As compared with placebo, Rivaroxaban increased the rates of major bleeding not related to coronary-artery bypass grafting (2.1% vs. 0.6%, *p* < 0.001) and also of intracranial hemorrhage (0.6% vs. 0.2%, *p* = 0.009), yet not increasing fatal bleeding (0.3% vs. 0.2%, *p* = 0.66) [72]. Despite the promising results showed in the ATLAS ACS 2-TIMI 51, Rivaroxaban 2.5 mg was not widely introduced in practice with this indication mostly for the safety concerns due to the higher bleeding risk and most importantly to the introduction of the more potent P2Y12 inhibitors prasugrel and ticagrelor which are now preferred over clopidogrel in patients with ACS. 

The APPRAISE-2 study tested the effect of Apixaban, at a dose of 5 mg twice daily in addition to standard antiplatelet therapy as compared with placebo in patients with a recent acute coronary syndrome and with high ischemic risk. The study was terminated prematurely after inclusion of 7392 patients because of an increase in clinically important bleeds, including fatal bleeds. The impact of apixaban on ischemic events could not be evaluated, because only 68% of the expected number of participants required to reach the pre-specified sample size was enrolled [73]. 

In the most recent Cardiovascular Outcomes for People Using Anticoagulation Strategies [74] trial, the role of factor Xa inhibition with Rvaroxaban was tested among chronic and stabilized patients with coronary artery disease (CAD) and in peripheral arterial disease (PAD) [74]. In this double-blind trial, the investigator randomly assigned 27,395 patients with stable atherosclerotic vascular disease to receive Rivaroxaban (2.5 mg twice daily) plus Aspirin (100 mg once daily), Rivaroxaban alone (5 mg twice daily), or Aspirin alone (100 mg once daily). The primary outcome was a composite of cardiovascular death, stroke, or myocardial infarction. The safety outcome was a modification of the International Society on Thrombosis and Haemostasis (ISTH) criteria for major bleeding and included fatal bleeding, symptomatic bleeding into a critical organ, bleeding into a surgical site requiring reoperation, and bleeding that led to hospitalization. With respect to the study primary endpoint, a treatment with rivaroxaban-plus-aspirin was associated to a significant 24% reduction of events as compared to aspirin-alone (379 patients vs. 496 patients; HR, 0.76; 95% CI, 0.66–0.86; *p* < 0.001), yet major bleeding were also more common in the rivaroxaban-plus-aspirin group (288 patients vs. 170 patients; HR, 1.70; 95% CI, 1.40–2.05; *p* < 0.001), mainly driven by nonfatal and non-intracranial bleedings. Rivaroxaban alone was not associated with lower rates of the primary endpoint compared with aspirin alone, but led to an excess of major bleeding events.

### 5.2. DOAC and Antiplatelet Therapy in Patients with Non-Valvular Atrial Fibrillation (AF) Undergoing PCI

**Figure 3:**
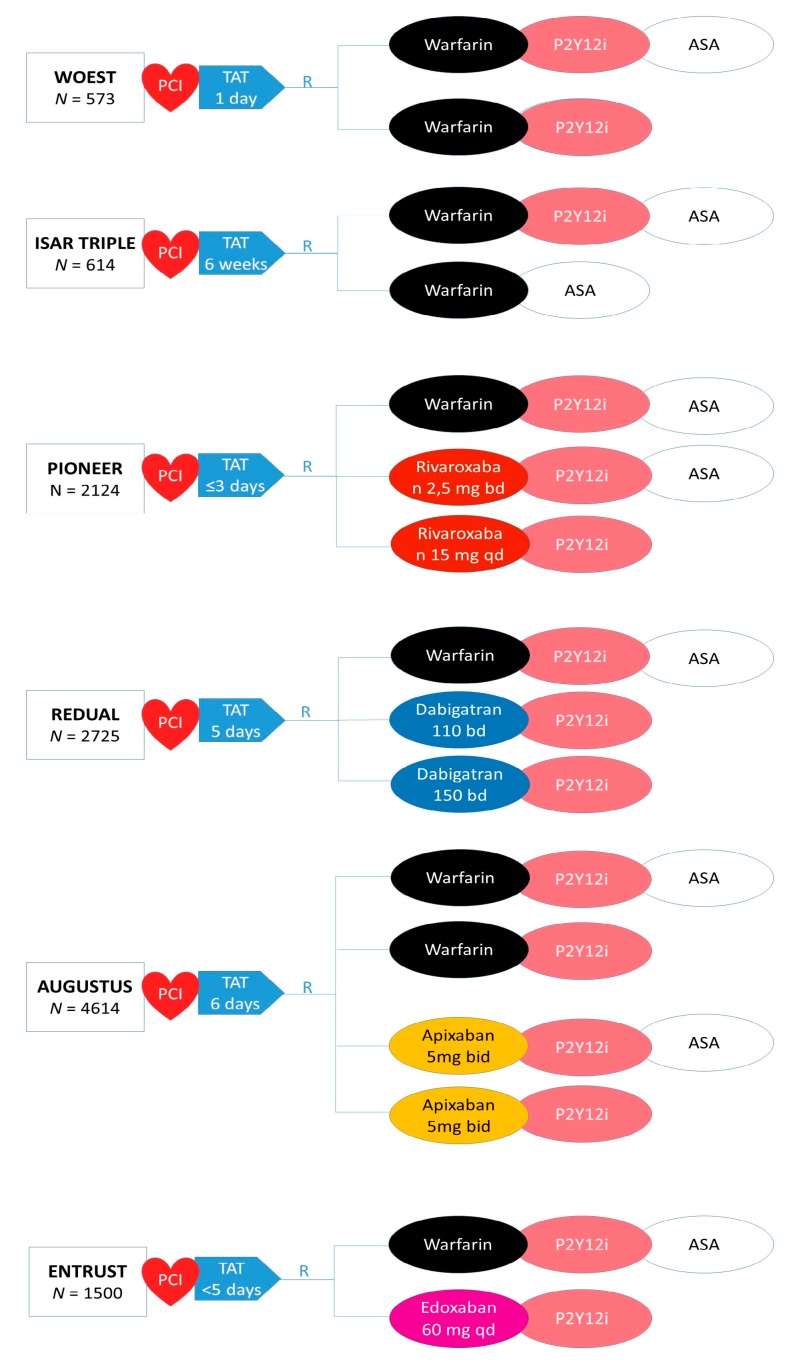
Design of clinical trials exploring the combination of oral anticoagulants and antiplatelet agents in patients with non-valvular atrial fibrillation undergoing percutaneous coronary intervention.

The association of anticoagulant and antiplatelet therapies is needed in the case of the concomitant presence of atrial fibrillation and percutaneous coronary intervention or recent acute coronary syndrome. Multiple studies demonstrated on one side that anticoagulant is inferior to antiplatelet therapy after stenting, and on the other side that antiplatelet therapy is inferior to anticoagulant therapy for the prevention of thromboembolic events in patients with atrial fibrillation [75]. However, the prolonged association of these two treatments expose patients to a hazard of serious bleeding, which is 3–4-fold higher than the two treatments taken singularly [76]. Hence, multiple studies focused on the reduction of duration or intensity of the antithrombotic therapy in this setting (Table 3 and Figure 3). The What is the Optimal antiplatElet and anticoagulant therapy in patients with OAC and coronary StenTing [77] WOEST trial randomized 573 patients with an indication to long-term oral anticoagulation (of whom 69% of patients had AF) and who underwent PCI to a dual therapy of oral anticoagulants vitamin k antagonists plus clopidogrel or to a triple therapy of OAC plus Clopidogrel and Aspirin. Treatment was continued for one month after bare metal stent (BMS) placement and for one year after DES placement (65% of patients). The primary endpoint was the occurrence of any bleeding episode at one-year follow-up. Dual as compared to triple therapy was associated with a substantial reduction of all bleeding events at one year (19.4 vs. 44.4%; HR 0.36, 95% CI 0.26–0.50; *p* < 0.001) and also to a reduction of the combined secondary endpoint of death, myocardial infarction, stroke, target-vessel revascularization, and stent thrombosis (11.1 vs. 17.6%; HR 0.56, 95% CI 0.35–0.91; *p* = 0.025) [77]. While the study was not powered to detect differences among rare ischemic events, a significant reduction of all-cause death was observed among patients treated with a dual therapy strategy. Nevertheless, this result should be taken with caution due to the open-label design of the study. More recently, multiple studies evaluated both the impact of aspirin withdrawal among patients with indication to triple therapy and the concomitant effect of DOAC vs. the traditional treatment with Vitamin k antagonists. The PIONEER AF-PCI was the first to study this scenario and randomized 2124 patients with non-valvular AF who had undergone PCI with stenting to receive, in a 1:1:1 ratio, low-dose rivaroxaban (15 mg o.d.) plus a P2Y12 inhibitor (and no ASA), very-low-dose rivaroxaban (2.5 mg b.i.d.) plus DAPT, or standard therapy with a dose-adjusted VKA plus DAPT. The primary study endpoint consisted of clinically relevant bleeding according to the TIMI definition. At 12 months, the rate of the primary endpoint was lower in the two groups receiving rivaroxaban than in the group receiving standard therapy (16.8% for rivaroxaban 15 mg + P2Y12i, 18.0% for rivaroxaban 2.5 mg + DAPT, and 26.7% for VKA + DAPT). The lowest rate of bleeding was observed among patients treated with rivaroxaban 15 mg + P2Y12 inhibitor, and this strategy was associated with a 41% reduction of bleeding as compared to triple therapy (HR 0.59, 95% CI 0.47–0.76; *p* < 0.001) [78]. Rates of ischemic events were similar in the three groups. While the lowest dosage of rivaroxaban is not approved for thromboembolic protection in AF, rivaroxaban 15 mg may be preferred over the 20 mg od dosage in patients recently treated with PCI and treated with concomitant antiplatelet therapy as endorsed by international guidelines [7]. 

Similarly, the Randomized Evaluation of Dual Antithrombotic Therapy with Dabigatran versus Triple Therapy with Warfarin in Patients with Nonvalvular Atrial Fibrillation Undergoing Percutaneous Coronary Interventio trial [79], randomized 2725 patients with AF who had undergone PCI to triple therapy with warfarin plus a P2Y12 inhibitor (clopidogrel or ticagrelor) and aspirin, or to dual therapy with dabigatran (110 mg or 150 mg twice daily) plus a P2Y12 inhibitor (clopidogrel or ticagrelor, at the discretion of the treating physician) and no aspirin (110-mg and 150-mg dual-therapy groups). Different from the PIONEER AF-PCI trial, the RE-DUAL PCI trial randomized patients to both the approved doses of dabigatran for thromboembolic protection in AF. In addition, the triple therapy duration in this study was much shorter than observed in the previous WOEST and PIONEER AF-PCI, in which a significant proportion of triple therapy patients continued treatment up to 12 months. The primary end point was a composite of major or clinically relevant nonmajor bleeding according to the ISTH definition. The RE-DUAL PCI trial was designed to test both the non-inferiority and the superiority of the experimental treatment (i.e., dual therapy with DOAC) for the primary bleeding endpoint and also its non-inferiority with respect to the incidence of a composite of thromboembolic events and coronary ischemic events (myocardial infarction, stroke, or systemic embolism, death, or unplanned revascularization). After a mean follow-up of 14 months, the primary endpoint occurred in 15.4% of patients in the dual-therapy group with dabigatran 110-mg as compared with 26.9% in the triple-therapy group (HR, 0.52; 95% CI, 0.42–0.63; *p* < 0.001 for both non-inferiority and superiority) and in 20.2% of patients in the dual-therapy group with dabigatran 150-mg as compared to 25.7% in the triple-therapy group (HR, 0.72; 95% CI, 0.58–0.88; *p* < 0.001 for non-inferiority). Non-inferiority for the composite ischemic endpoint was also confirmed with both doses of dabigatran. 

On the same line as in the previous two trials, the antithrombotic Therapy after Acute Coronary Syndrome or PCI in Atrial Fibrillation (AUGUSTUS) study evaluated the impact of a treatment with apixaban as compared to VKA in patients undergoing stenting or with a recent ACS. Different from the previous three studies, this used a two-by-two factorial design, i.e. not only the type of oral anticoagulant but also aspirin treatment was randomized. AUGUSTUS is thus the first trial to test both the concept of OAC type and dual vs. triple therapy separately. In total, 4614 patients were randomly allocated to apixaban 5 mg bid or VKA, and to aspirin or placebo, on top of a P2Y12 inhibitor (mostly clopidogrel) after a mean of seven days after the PCI/ACS occurrence. At six-month follow-up, the primary outcome of major or clinically relevant nonmajor bleeding was reported in 10.5% of the patients receiving apixaban, as compared with 14.7% of those receiving a vitamin k antagonist (HR, 0.69; 95% CI, 0.58–0.81; *p* < 0.001 for both non-inferiority and superiority) reaching both the prespecified non-inferiority and superiority of the experimental treatment. With respect to the second randomization, the primary endpoint occurred in 16.1% of those assigned to aspirin, as compared to 9.0% with placebo (HR, 1.89; 95% CI, 1.59–2.24; *p* < 0.001). A lower incidence of death or hospitalization was observed in patients treated with apixaban compared to vitamin k antagonists (23.5% vs. 27.4%; hazard ratio, 0.83; 95% CI, 0.74–0.93; *p* = 0.002). With respect to the secondary endpoint exploring ischemic events, this was similar among patients treated with apixaban or VKA and also for patients randomized to aspirin or placebo. Ischemic stroke was significantly reduced by 50% among patients in the apixaban arm. Despite the fact that this study was not powered to evaluate differences among study arms for the rare ischemic endpoints, a trend towards an excess of myocardial infarction and definite or probable stent thrombosis was observed among patients randomized to placebo (dual therapy) as compared to those assigned to aspirin (triple therapy) [80]. 

Finally, the recent Edoxaban Treatment Versus Vitamin K Antagonist in Patients With Atrial Fibrillation Undergoing Percutaneous Coronary Intervention (ENTRUST-AF-PCI) evaluated the safety and efficacy of edoxaban plus single antiplatelet therapy vs. VKA plus DAPT in subjects with atrial fibrillation following PCI with stent placement. Participants were randomly assigned (1:1) from four hours to five days after PCI to edoxaban (60 mg once daily) plus a P2Y12 inhibitor (mostly clopidogrel) for 12 months or a VKA in combination with a P2Y12 inhibitor and aspirin (100 mg once daily, for 1–12 months). The edoxaban dose was reduced to 30 mg per day if one or more factors were present (creatinine clearance 15–50 mL/min, bodyweight ≤60 kg, or concomitant use of specified potent *p*-glycoprotein inhibitors). The primary endpoint was a composite of major or clinically relevant non-major (CRNM) bleeding within 12 months. In the intention-to-treat analysis, major or CRNM bleeding events occurred in 128 (17%) patients randomized to Edoxaban and 152 (20%) patients to the VKA regimen; the relative risk reduction of 17% for the safety endpoint was enough to demonstrate the non-inferiority of the experimental treatment, but did not reach superiority (95% CI 0.65–1.05; *p* = 0.0010 for non-inferiority, *p* = 0.12 for superiority). In a post-hoc landmark analysis in which events occurring during the first two weeks after randomization were censored, experimental treatment with Edoxaban was associated to a significant 32% reduction of bleeding compared to triple therapy with VKA. No difference for ischemic events was observed, yet the study was not powered to detect differences for ischemic endpoints [81]. As already reported, none of these RCTs taken singularly was powered for ischemic endpoints, hence meta-analysis could provide important insights on this matter. However, the meta-analysis currently available in the literature drafted different conclusions. A first study-level network meta-analysis of four RCT and 10,026 patients, not including the more recent data from the ENTRUST trial, showed that dual therapy with both VKA or DOAC was associated to lower TIMI major or minor bleeding compared to triple therapy with VKA, and a similar risk of MACE, MI and ST [82]. On the contrary, a more recent patient-level meta-analysis including the recent results of the ENTRUST trial, showed a trend (although not significant) towards higher risk for stent thrombosis with dual therapy. Reconciling these inconsistencies will be a priority of future research to inform international practice guidelines. Ideally, more robust data may come from patient-level meta-analysis, which could also provide important insights on the subgroups that most benefit from each treatment. 

Leveraging on the new evidence in the field, in particular the REDUAL-PCI and PIONEER-AF-PCI, a recent North American consensus statement on the management of antithrombotic therapy in patients with atrial fibrillation undergoing percutaneous coronary intervention [83] suggests a double-therapy approach as the default strategy for most patients. Instead, in selected patients at high ischemic/thrombotic and low bleeding risks, extending low-dose aspirin therapy (triple therapy) up to one month after PCI appear reasonable. In addition, in this setting, clopidogrel remains the P2Y12I of choice. 

Finally, whether antiplatelet therapy should be stopped 12 months after PCI, leaving patients on treatment with OAC alone was a matter of debate. The recent Atrial Fibrillation and Ischemic Events with Rivaroxaban in Patients with Stable Coronary Artery Disease (AFIRE) trial compared rivaroxaban monotherapy compared with rivaroxaban/antiplatelet therapy after 12 months from PCI or CABG and up to 24 months in patients with atrial fibrillation and stable coronary artery disease. The study was prematurely interrupted for an excess of mortality in the combination therapy group treated with rivaroxaban plus antiplatelet therapy. The primary efficacy outcome of all-cause mortality, myocardial infarction, stroke, unstable angina requiring revascularization, or systemic embolism, occurred in 4.1%/patient-year in the rivaroxaban monotherapy group compared with 5.8%/patient-year in the rivaroxaban/antiplatelet therapy group (*p* for non-inferiority <0.0001). As expected, ISTH major bleeding occurred in 1.6%/patient-year in the rivaroxaban monotherapy group and in 2.8%/patient-year in the rivaroxaban/antiplatelet therapy group (*p* = 0.01). 

## 6. Current Evidence for the Type and Duration of Antithrombotic Therapy in Percutaneous Structural Heart Interventions

### 6.1. Transcatheter Aortic Valve Implantation (TAVI)

Transcatheter Aortic Valve Implantation (TAVI) has been established as the method of choice for the treatment of severe aortic stenosis in patients with increased surgical risk [84,85]. Due to the vibrant activity in clinical trials, which is extending the indications of this treatment to different and lower risk clinical indications, TAVI could become the treatment of choice for most patients with aortic valve disease [86]. Secondary prevention with antithrombotic therapies after TAVI is primarily meant to reduce the risk of thrombosis of the valve components and prevent subsequent systemic thrombus embolization. While most of the events occurs during the first 48 h after valve implantation and are likely related to acute embolization of fibro-calcific valve material after valve implantation or catheter manipulation potentially damaging aortic wall, later ischemic events may be linked to thrombosis of the prosthesis surface or to unrecognized/new-onset of atrial fibrillation [87]. 

**Figure 4:**
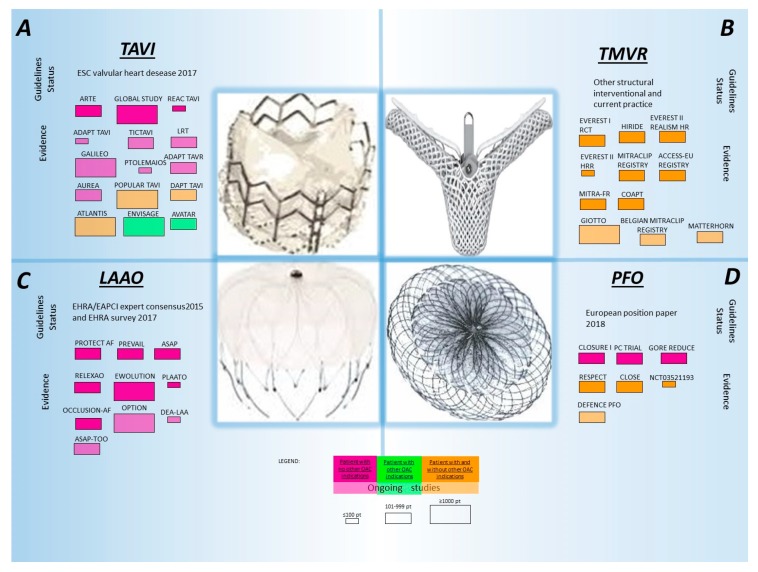
Clinical studies testing antithrombotic strategies for secondary prevention of percutaneous structural heart interventions. TAVI: transcatheter aortic valve implantation; TMVR: transcatheter mitral valve repair; LAAO: left atrial appendage occlusion; PFO: patent foramen ovale.

Current guidelines, mostly based on expert consensus extrapolated from the coronary stent experience, suggest as standard treatment strategy DAPT with aspirin and clopidogrel for 3–6 months after valve implantation, followed by a lifelong treatment with ASA. However, in patients with low hemorrhagic risk, a therapy with vitamin k antagonists in the first months after the procedure is also considered reasonable [84]. This latter indication is extrapolated from the experience with surgically-implanted valves, in which a short-term anticoagulation after bioprothetic valve implantation is common [84]. Despite the paucity of high-quality evidence in the field, several studies are evaluating possible antithrombotic treatment strategies after TAVI (Figure 4).

In the randomized Aspirin Versus Aspirin + Clopidogrel Following Transcatheter Aortic Valve Implantation Trial, 222 TAVI patients undergoing a balloon-expandable device implantation were randomized to DAPT (i.e., aspirin plus clopidogrel) or to aspirin alone. The primary endpoint of death, MI, stroke or transient ischemic attack, or major or life-threatening bleeding was similar between the two treatment groups but trended higher among patients assigned to DAPT (15.3% vs. 7.2%, *p* = 0.065) mostly due to an excess of major or life-threatening bleeding among patients treated with DAPT (10.8% vs. 3.6%, *p* = 0.038) [88]. Sherwood et al. tried to describe contemporary practice patterns of antiplatelet therapy and their relationship to outcomes post-TAVI trough a retrospective analysis of the NCDR STS/ACC TVT Registry. This analysis of 16,694 patients undergoing transfemoral TAVI reported that 81.1% patients were discharged on DAPT and 18.9% were discharged on SAPT. There was no difference in the main baseline characteristics among those assigned to DAPT or SAPT, whereas those assigned to DAPT had more CAD (64.6% vs. 52.3%; *p* < 0.01) and PAD (25.2% vs. 22.3%; *p* < 0.01). No difference in mortality (HR 0.92; 95% CI 0.81, 1.05) stroke (HR 1.04; CI 0.83, 1.31) or MI (HR 1.00; CI 0.72, 1.39) at one year was observed between DAPT and SAPT, but a significantly higher risk for major bleeding (HR 1.48; CI 1.10, 1.99) was observed with DAPT [89]. Current TAVI population carries an exaggerated bleeding risk, hence more potent platelet inhibition has to be justified by other concomitant indications rather that procedural alone. Whether the type and not only the number of the antiplatelet drugs implemented may have an impact in patients with TAVI is under investigation in the TICTAVI study (NCT02817789), which will evaluate the impact of ticagrelor monotherapy for 30 days after valve implantation vs. DAPT with clopidogrel and the soluble salt of aspirin lysine Acetylsalicylate. 

Apart from antiplatelet therapy, various regimens of anticoagulant therapy are currently under investigation for secondary prevention after TAVI in patients without further indication to OAC. Based on the fact that valve thrombosis may be associated to a low shear stress thrombosis and that the current TAVI population has a high risk of new onset AF which was registered in up to 50% after valve implantation [90], use of OACs in this population may appear sound. The Global Study compares a rivAroxaban-based Antithrombotic Strategy with an antipLatelet-based Strategy After Transcatheter aortIc vaLve rEplacement to Optimise Clinical Outcomes (GALILEO) trial (NCT02556203) compared rivaroxaban 10 mg combined with aspirin for three months followed by rivaroxaban 10 mg monotherapy versus DAPT for three months followed by aspirin monotherapy in patients without an established indication for OAC [91]. The trial has been prematurely terminated due to an excess of death or thromboembolic events (11.4% vs. 8.8%), bleeding (4.2% vs. 2.4%) and all-cause death (6.8% vs. 3.3%) in patients allocated to rivaroxaban. Similarly, the ongoing DAPT versus OAC for a Short Time to Prevent Cerebral Embolism After TAVI (AUREA) trial (NCT01642134) will compare VKA with DAPT in patients without further indication to OAC. The primary study endpoint is the detection of new areas of cerebral infarction by MRI three months after TAVI. Further ongoing studies are evaluating the safety and efficacy of DOACs in patients undergoing TAVI. The ATLANTIS (Anti-Thrombotic Strategy After Trans-Aortic Valve Implantation for Aortic Stenosis trial) (NCT02664649) is testing the safety and efficacy of apixaban 5 mg bid compared to VKA in patients with an established indication for OAC, and apixaban 5 mg bid compared to DAPT/SAPT in patients without an indication for OAC. Similarly, the ENVISAGE AF trial (NCT02943785) is testing Edoxaban 30 and 60 mg vs. VKA in patients with AF and an indication for OAC.

Apart from secondary prevention of thromboembolic events, the evidence of clinically-evident valve thrombosis after biologic valve implantation has been described and require specific treatment to reduce the risk of embolization or prosthesis dysfunction. Prosthetic valve thrombosis is characterized by thrombus formation on the leaflets and metallic frames, with subsequent valve dysfunction with or without thromboembolism. Latib et al. reported in 4266 patients undergoing TAVI a total of 26 cases of PV thrombosis (0.61%) at a median timing from implantation of 181 days (interquartile range: 45–313 days) [92]. The most common clinical findings in these cases were presentation with progressive dyspnea, signs of heart failure or systemic embolization. Valve thrombosis could also be an incidental finding at the time of echocardiographic follow-up, detected by an increase transprosthetic gradient [93]. Valve thrombosis after TAVI could be acute (up to one day), subacute (ten days to one month), or late (more than one month) and its risk appears higher in the first three months post-implantation. Although the mechanisms are not completely clear, risk factors such as systemic pro-thrombotic state, valve malposition, prosthesis size, and the presence of low/high velocity flows after implantation [94] have been suggested as potential risk factors for this phenomenon. In addition, subclinical valve thrombosis has also been describing using novel imaging techniques but its clinical implications are still unclear. Makkar et al. showed that in patients with stroke after valvular implantation there was a reduction in the mobility of leafleats identifiable by CT scan as a hypo-attenuation on the valve leaflets after administration of contrast medium. In all cases, the reduction of leaflet motion was not related to an alteration of the valvular hemodynamics but seems to be related with an in increased risk of TIA/stroke (in the analysis of pooled RESOLVE and SAVORY cohorts). A treatment with OAC resolved leaflet hypomobility in all the patients treated, while other antithrombotic regimens did not prove effective [95]. Several observational studies showed a lower risk of valve thrombosis in patients on OAC treatment [96,97]. Latib et al. showed that, of 26 patients with valve thrombosis, 23 (88%) were treated with medical therapy, such as oral vitamin k antagonists with/without bridging heparin (unfractionated heparin or low-molecular–weight heparin). Anticoagulation was effective and resulted in significant decrease of the trans-prothesic aortic valve gradient or disappearance of the thrombotic mass in all patients [92]. The upcoming Comparison of a Rivaroxaban-based Strategy With an Antiplatelet-based Strategy Following Successful TAVR for the Prevention of Leaflet Thickening and Reduced Leaflet Motion as Evaluated by Four-dimensional, Volume-rendered Computed Tomography (GALILEO-4D) (NCT02833948) will also evaluate if anticoagulation compared to the usual double platelet inhibitor therapy after TAVRI may have an impact in reducing the risk of leaflet thrombosis, and its results are expected soon.

### 6.2. Evidence for MitraClip, Transcatheter Mitral Valve Interventions

Transcatheter mitral valve repair is indicated in moderate-to-severe or severe mitral insufficiency in patients with high surgical risk [98]. The MitraClip is currently the only system approved by the FDA and is based on the concept of “edge to edge” surgical repair to reduce valve insufficiency [99]. The procedure involves the passage of large bore catheter from the venous system, through atrial septal puncture in the left atrium, which may expose patients to peri-procedural and post-procedural ischemic events [100]. An incidence of 0.9% of ischemic stroke was documented on 30-day follow-up in the EVEREST RCT trial [98], 2.6% in the EVEREST-HRR and 2.4% in the EVEREST-REALISM registries [101] (Figure 4). Currently, neither international guidelines nor manufacturers provide precise recommendations for the type and duration of the antithrombotic therapy after the procedure and no specific studies have been yet carried out in this field. The current most commonly adopted antithrombotic treatments derive from the protocol recommendations of the first pivotal trials of the device. This generally consist of one month of DAPT with aspirin and clopidogrel followed by aspirin alone for 6–12 months. Transcatheter mitral valve replacement is flourishing and it is still at its inception. Multiple devices have been tested in first-in-man studies and the approach to antithrombotic therapy is still anecdotic and should be individualized based on the specific device used. Given the slow blood flow on the atrial side of the prosthesis together with the high rate of AF in these patients, the risk of device thrombosis is potentially high and merits consideration at the time of antithrombotic treatment selection. 

### 6.3. Percutaneous Patent Foramen Ovale (PFO) Occlusion

Patent foramen ovale (PFO) and atrial septal defects [8] represent the most prevalent cardiac anomaly in the general population [102,103]. The presence of PFOs may predispose to paradoxical right-to-left atrium embolism, of which the most dreadful consequence is cryptogenic stroke, representing a major cause of embolic stroke in younger patients. Several devices have been developed in the last 20 years to physically occlude the PFO and prevent right-to-left shunt embolism [104,105] and systemic embolization. While these devices are effective for the scope, several procedural and long-term complications exist, including atrial rupture, pericardial tamponade, septal perforation, and acute/late thrombus formation of the device [106]. Thrombus formation can be a consequence of the incorrect measurement of the device [107,108]. More typically thrombosis could be triggered by the metal structure of the device, owing to endothelialization defects within the first four weeks [109]. However experimental studies in vivo have shown that the endothelialization process can last up to three months [110], or even up to five years post implantation [111] in some reports. Independent predictors of thrombotic formation are atrial fibrillation and presence of septal aneurysm [109]. Antithrombotic therapy is therefore crucial after device implantation, but the strategies remain controversial, and no randomized studies have been published to assess the optimal antithrombotic strategy in this scenario (Figure 4). In many centers, the antithrombotic strategy of choice is DAPT with ASA plus clopidogrel for 6–8 weeks followed by ASA alone for an additional 4–8 months. DAPT for 1–6 months followed by single antiplatelet therapy for at least five years is recommended in a recent consensus document endorsed by the EAPCI [112]. In contrast, the latest American Academy of Neurology guidelines recommended lifelong antithrombotic therapy after PFO closure [113]. In the REDUCE trial, 664 patients with previous cryptogenic stroke were randomized in a 2:1 ratio to undergo PFO closure with the Gore Occluder. Antiplatelet therapy consisted of ASA alone (59%), clopidogrel alone (25.9%) or a combination of aspirin and P2Y12 inhibitor (i.e., clopidogrel or dypiridamole, <10%) and was similar in the two study groups. Follow-up was continued during a median of 3.2 years after the procedure, and in this period antiplatelet was maintained. The risk of recurrent stroke was significantly lower with PFO closure plus antiplatelet therapy than with antiplatelet therapy alone (1.4% vs. 5.4%, *p* = 0.002) [114]. Device thrombosis was reported in two patients in the closure group. In the CLOSE trial, 663 patients with a recent stroke attributed to PFO were assigned in a 1:1:1 ratio to PFO closure (11 different devices were used) plus long-term antiplatelet therapy, oral anticoagulation alone or antiplatelet therapy alone. The PFO closure group received DAPT for three months, followed by SAPT for a median follow-up of 5.5 years. Among patients assigned to oral anticoagulation, 93% received VKA and 7% DOACs. In the antiplatelet therapy group, 87% received ASA alone, 10% Clopidogrel alone and 3% ASA plus dipyridamole throughout the study period. At 12-month echocardiography evaluation, 93% of patients who underwent closure had no or minimal residual shunt. The rate of recurrent stroke at five years was significantly lower with PFO closure plus long-term antiplatelet therapy than with antiplatelet therapy alone (0% vs. 4.9%, *p* < 0.001) [115]. Within the comparison of antiplatelet therapy or anticoagulant therapy, recurrent stroke at five years was observed in 3.8% of patients assigned to antiplatelet therapy vs. 1.5% of patients assigned to oral anticoagulant therapy, however statistical significance was not analyzed because the study was not powered to compare outcomes in these groups, hence any conclusion regarding the indirect comparison of PFO closure and anticoagulant therapy cannot be extrapolated from this study. One device thrombosis was reported, and no significant differences in terms of major bleeding were recorded. In the RESPECT trial, 980 patients with previous cryptogenic ischemic stroke were randomly assigned to undergo PFO closure (Amplatz PFO occluder) or receive medical therapy for a median follow-up of 5.9 years. Patient undergoing PFO closure received ASA plus clopidogrel daily for one month, followed by ASA alone for five months. In the medical-therapy group, four regimens were allowed: ASA alone, clopidogrel alone, warfarin with a goal INR of 2–3, and ASA plus dipyridamole. After a median follow-up of 5.9 years, closure of PFO was associated with a lower rate of recurrent ischemic stroke than medical therapy (3.6% vs. 5.8%, *p* = 0.007). There were two cases of device thrombosis treated successfully with intravenous heparin. Wintzer-Wehekind et al. in a retrospective analysis 453 patient after PFO closure reported a low but clinically relevant risk of bleeding, which exceeded the risk of ischemic events in their cohort. As expected, all major bleeding events occurred in patients receiving antiplatelet therapy, and only one fifth of the patients stopped the antithrombotic therapy within one year after PFO closure. Antiplatelet therapy discontinuation was not associated with any increase in ischemic events. The authors concluded that, in patients without any other risk factors, shorter-term (≤1 year) antiplatelet treatment after PFO closure is a safer option [116]. 

### 6.4. Left Atrial Appendage Occlusion (LAAO)

Imaging studies have shown that 90% of thrombotic formations detected in patients with non-valvular atrial fibrillation are located in the left atrial appendage [117,118]. For this reason, percutaneous LAA occlusion (LAAO) has become an alternative to anticoagulation for patients that are ineligible or contraindicated to the medical treatment [119]. 

The first case of LAA surgical occlusion was reported in 1949 [120], while the first device for percutaneous exclusion was the PLAATO [121]. This was followed by introduction of the Watchman device (Boston Scientific) in 2002 and the Amplatzer cardiac plug in 2008, which are currently the most used devices in clinical practice. Watchman remains the only device studied in properly sized randomized trials, such as PROTECT AF [122] and the PREVAIL [123]. 

The device-oriented need for antithrombotic therapy after percutaneous LAAO has been tackled differently in various randomized studies and is meant to reduce the risk of device thrombosis, which is associated with an increased risk of stroke (Figure 4). Device thrombosis was observed in 4.2% of patients in the PROTECT AF trial with a stroke rate attributable to this event of 0.6%. Device thrombosis in other recent registries ranged from 1.3% to 7.2% [124]. While most of the data currently available are obtained from the Watchman device, there is currently a similar approach to antithrombotic therapy irrespective of the type and size of device implanted. Complete device endothelization has been observed after 90 days in animal models, but this time may be protracted in humans, and interindividual variability may be significant, adding uncertainty regarding the optimal type and duration of the antithrombotic therapy after LAAO. In the PROTECT AF trial, which included patients with AF without concomitant high bleeding risk, VKA with a target INR of 2–3 was implemented for 45 days after LAAO and was followed by DAPT for six months and then lifelong aspirin monotherapy. The same approach was used in the PREVAIL trial [123]. A different approach has been used in registries including high bleeding risk patients. The ASAP study included patients with an absolute contraindication to oral anticoagulation, and in this setting the protocol mandated six months of DAPT followed by lifelong aspirin [122]. Much more variability have been observed in recent real-world registries, which reflect the extreme heterogeneity of this patient population often requiring a personalized approach. In the EWOLUTION registry for example, antithrombotic regimens after LAAO were as follows: warfarin in 16%, DOAC in 11%, DAPT in 60%, SAPT in 7%, and no therapy in 6% [125]. In a survey by European Heart Rhythm association, showed that DAPT for six weeks to six months followed by aspirin monotherapy was the most common regimen, while 41% of centers would prescribe no therapy after LAAO and less than 10% would follow the antithrombotic regimen advocated by the PROTECT AF protocol [126]. 

While a randomized comparison on the efficacy of different antithrombotic strategies is lacking, a recent propensity matched analysis of 1527 patients from various trials and registries implementing the WATCHMAN device compared OAC vs. antiplatelet therapy after LAAO. The OAC arm included patients treated with OAC for 45 days after device implantation followed by six months of single or dual antiplatelet therapy. The antiplatelet therapy arm included patients managed with antiplatelet therapy alone, either dual or single, for various durations. While the rate of hard endpoints, including bleeding and thromboembolic events, was similar between the two treatment groups, a significant excess of device thrombosis was observed in the group among managed with antiplatelet therapy alone (OAC 1.4% vs. APT 3.1%; *p* = 0.018). While this comparison was not randomized and there was a high heterogeneity of treatment types and duration among the two study arms (e.g., APT included both DAPT and SAPT for various duration), this study stressed the importance of specific antithrombotic treatment after LAAO. In a retrospective series of 487 patients undergoing WATCHMAN or ACP/Amulet implantation, 208 received SAPT therapy only or no antithrombotic therapy after LAAO. In this series device thrombosis was as high as 7.2%, and in multivariable analysis DAPT or OAC therapy were protective for device thrombosis compared to SAPT or no therapy, raising concerns on the safety of such treatment strategy after LAAO [124]. 

While awaiting future randomized clinical trials which are urgently needed to shed light on the optimal antithrombotic treatment strategy after LAAO, the European Heart Rhythm Association/European Association of Percutaneous Cardiovascular Interventions (EHRA/EAPCI) expert consensus statement recommends treatment with OAC for at least six weeks followed by DAPT for six months and a single antiplatelet drug thereafter in patients without contraindications for OAC. Instead, patients ineligible to OAC should be managed with DAPT for 1–6 month followed by aspirin monotherapy indefinitely [125]. In addition, evidence coming from real-world registries, supported the safety of treatment with DOAC post-LAAC, and updates in international LAAC device labeling now admits three-month DAPT or direct OAC therapy post-LAAC, if the standard regimen with 45 days of warfarin followed by DAPT up to six months is not an option.

Importantly, since the risk of device thrombosis varies based on both patients’ characteristics and device placement, personalization of the treatment type and duration taking into consideration these variables together with the baseline bleeding risk should always be a priority. The most recognized predictors of stroke and device thrombosis are left ventricular ejection fraction <40%, female sex, smoking, higher CHA2DS2-VASc score, spontaneous echocardiographic contrast, pre-existing LAA thrombus, and LAA peak emptying velocity [127,128,129]. Other mechanical factors such as deep implantation of the device forming a neoappendage or failure of disc apposition and significant residual device leak (>5 mm) are other factors of additional thromboembolic risk.

## 7. Conclusions

Antithrombotic agents are among the most prescribed treatments in cardiology, and their implementation for secondary prevention of spontaneous vascular or device-related thrombotic complications after percutaneous cardiovascular interventions has been extensively explored. While dual antiplatelet therapy after PCI and stenting has been studied in the last two decades, the search for the optimal antithrombotic therapy after structural interventions is still in its infancy. Intensification or prolongation of the antithrombotic strategy is generally associated with a superior efficacy for ischemic events prevention but also to an excess of bleeding, which should be avoided. Patient-centered data informing decision-making and treatment personalization represent the main objective of future research, in order to optimize the balance between ischemic and bleeding complications.

## Figures and Tables

**Table 1:** Randomized controlled trials of dual antiplatelet therapy duration in patients treated with percutaneous coronary intervention.

	Year	*N*	Study Design	Population Type	Type of Stent	Type of P2Y12 Inhibitor	DAPT Duration	Primary Endpoint	Event Rates (Short vs. Long)	Duration of Follow-Up
**CREDO**	2002	2116	Superiority of 12 mo DAPT *	ACS 67%	BMS 76.3% POBA 23.7%	Clopidogrel 100%	1 vs. 12	Death MI or stroke	11.5% vs. 8.5%	12 mo
**PRODIGY**	2012	1970	Superiority of 24 mo DAPT	ACS 75%	BMS 25% 1st gen DES 25% 2nd gen DES 50%	Clopidogrel 100%	6 vs. 24	All-cause death, MI, CVA	10% vs. 10.1%	24 mo
**ARTIC** **INTERRUPTION**	2012	1259	Superiority of 12 mo DAPT	ACS 34%	1st gen DES 41% 2nd gen DES 63%	Clopidogrel 91% Prasugrel 9%	12 vs. 18–24	All-cause death, MI, ST, stroke, TVR	4% vs. 4%.	17 mo
**EXCELLENT**	2012	1443	Non-inferiority of 6 mo DAPT	ACS 51%	1st gen DES 25% 2nd gen DES 75%	Clopidogrel 100%	6 vs. 12	Cardiac death, MI, TVR	4.8% vs. 4.3%	12 mo
**RESET**	2012	2117	Non-inferiority of 3 mo DAPT	ACS 59%	1st gen DES 21% 2nd gen DES 85%	Clopidogrel 100%	3 vs. 12	Cardiac death, MI, ST, TVR, major bleeding	4.7% vs. 4.7%	12 mo
**OPTIMIZE**	2013	3119	Non-inferiority of 3 mo DAPT	ACS 35%	2nd gen DES 100%	Clopidogrel 100%	3 vs. 12	All-cause death, MI, stroke, major bleeding	6% vs. 5.8%	12 mo
**DES-LATE**	2014	5045	Superiority of 24 mo DAPT	ACS 61%	1st gen DES 64% 2nd gen DES 30%	Clopidogrel 100%	12 vs. 36	Cardiac death, MI, stroke	2.4% vs. 2.6%	24 mo
**DAPT**	2014	9961	Superiority of 30 mo DAPT	ACS 43%	1st gen DES 38% 2nd gen DES 60%	Clopidogrel 65.3% Prasugrel 34.7%	12 vs. 30	Death, MI, stroke, ST.	7.3% vs. 4.7%	33 mo
**SECURITY**	2014	1399	Non-inferiority of 6 mo DAPT	28% ACS	2nd gen DES 100%	Clopidogrel 98.7% Prasugrel 0.2% Ticagrelor 0.4%	6 vs. 12	Cardiac death, MI, ST, or stroke	4.5% vs. 3.7%	12 mo
**ISAR SAFE**	2015	4000	Non-inferiority of 6 mo DAPT	ACS 40%	1st gen DES 10% 2nd gen DES 89%	Clopidogrel 100%	6 vs. 12	Death, MI, ST, stroke, major bleeding	1.5% vs. 1.6%	15 mo
**ITALIC**	2015	1822	Non-inferiority of 6 mo DAPT	ACS 24%	2nd gen DES 100%	Clopidogrel 98.6% Prasugrel 1.7% Ticagrelor 0.05%	6 vs. 24	Death, MI, TVR, stroke, major bleeding	1.6% vs. 1.5%	24 mo
**I LOVE IT 2**	2016	1829	Non-inferiority of 6 mo DAPT	ACS 64%	2nd gen DES 100%	Clopidogrel 100%	6 vs. 12	Cardiac death, target vessel MI	7.5% vs. 6.3%	18 mo
**OPTIDUAL**	2016	1385	Superiority of 48 mo DAPT	ACS 36%	1st gen DES 34% 2nd gen DES 59%	Clopidogrel 100%	12 vs. 48	Death, MI, stroke, ISTH major bleeding	7.5% vs. 5.8%	33 mo after randomization
**IVUS XPL**	2016	1400	Comparability of 6 vs. 12 mo DAPT	ACS 49%	2nd gen DES 100%	Clopidogrel 100%	6 vs. 12	Cardiac death, MI, stroke, or major bleeding	2.2% vs. 2.1%	12 mo
**NIPPON**	2016	3307	Non-inferiority of 6 mo DAPT	ACS 33%	2nd gen DES 100%	Clopidogrel 97.5% Prasugrel 0.1% Ticlopidine 2.3%	6 vs. 18	Death, MI, CVA, major bleeding	2.1% vs. 1.5%	18 mo
**REDUCE**	2017	1496	Non-inferiority of 3 mo DAPT	ACS 100%	2nd gen DES 100%	Clopidogrel 40.8% Prasugrel 10.4% Ticagrelor 48.9%	3 vs. 12	All-cause death, MI, ST, stroke, TVR, or bleeding	8.3% vs. 8.5%	12 mo
**DAPT-STEMI**	2017	861	Non-inferiority of 6 mo DAPT	ACS (STEMI) 100%	2nd gen DES 100%	Clopidogrel 42.0% Prasugrel 29.5% Ticagrelor 28.5%	6 vs. 12	All-cause mortality, MI, revascularization, stroke, and TIMI major bleeding	4.8% vs. 6.6%	24 mo
**OPTIMA-C**	2018	1368	Non-inferiority of 6 vs. 12 mo DAPT	ACS 50%	2nd gen DES 100%	Clopidogrel 100%	6 vs. 12	Cardiac death, TVR MI, Ischemia-driven TVR	1.2% vs. 0.6%	12 mo
**SMART-DATE**	2018	2712	Non-inferiority of 6 mo DAPT	ACS 100%	2nd gen DES 100%	Clopidogrel 80.7% Prasugrel/ Ticagrelor 19.3%	6 vs. 12	All-cause mortality, MI, stroke	4.7% vs. 4.2%	18 mo
**GLOBAL LEADERS**	2018	15,968	Superiority of 1 mo DAPT followed by 23 mo ticagrelor monotherapy vs. 12 mo DAPT followed by 12 mo ASA	ACS 47%	2nd gen DES 100%	Ticagrelor 46.8% Clopidogrel 53.2%	1 vs. 12	All-cause mortality non-fatal Q-wave MI	3.8% vs. 4.3%	24 mo
**STOP DAPT 2**	2019	3045	Non-inferiority of 1 month of DAPT followed by clopidogrel monotherapy compared with 12 mo DAPT	ACS 38.2%	2nd gen DES 100%	Clopidogrel 100%	1 vs. 12	CV death, MI, ischemic or hemorrhagic stroke, definite ST, or major or minor bleeding	2.4% vs. 3.7%	12 mo
**SMART CHOICE**	2019	2993	Non-inferiority of 3 mo of DAPT followed by P2Y12 inhibitor monotherapy compared with 12 mo DAPT	ACS 58.2%	2nd gen DES 100%	Clopidogrel 77.2% Prasugrel or Ticagrelor 22.8%	3 vs. 12	Death, MI or stroke	2.9% vs. 2.5%	12 mo

* The 12-month DAPT arm was associated to 300 mg clopidogrel loading dose before PCI, whereas the one-month DAPT arm was associated to placebo loading dose before PCI; ACS, acute coronary syndrome; BMS, bare metal stent; CV, cardiovascular; CVA, cerebrovascular accident; DAPT, dual antiplatelet therapy; DES, drug eluting stent; ISTH, International Society of Thrombosis and Hemostasis; MI, myocardial infarction; MO, months; POBA, plain old balloon angioplasty; ST, stent thrombosis; TIMI, The Thrombosysis in Myocardial Infarction; TVR, target vessel revascularization.

**Table 2:** Randomized controlled trials evaluating the effect of commercially available direct oral anticoagulants in patients with coronary artery disease.

	Randomization	*N*	Study Type	Recent ACS%	Age	Type of Antiplatelet Therapy Associated	F.U	Study Hypothesis	Primary Efficacy Endpoint	Primary Safety Endpoint	Conclusion
**RE-DEEM**	Dabigatran 50 mg bid 75 mg bid 110 mg bid 150 mg bid vs. Placebo	1861	Double Blind Phase 2	100%	61.8	Aspirin + Clopidogrel	6 month	Explore the rate of bleeding with dose escalating Dabigatran triple therapy	Reduction in D-dimer levels	Major or clinically relevant minor bleeding (ISTH, TIMI e GUSTO)	Dose-dependent increase in bleeding events with Dabigatran on top of antiplatelet therapy
**ATLAS** **ACS-TIMI 46**	Rivaroxaban 5 mg od (or 2.5 mg bid) or 20 mg od (or 10 mg bid) vs. Placebo	3491	Double Blind Phase 2	100%	58	Aspirin Alone or Aspirin + Thyenopiridine	6 month	Explore the rate of bleeding with dose escalating Rivaroxaban triple/dual therapy	Death, myocardial infarction, stroke, or severe recurrent ischemia requiring revascularization	TIMI major, TIMI minor, or requiring medical attention	Dose-dependent increase in bleeding events with Rivaroxaban on top of antiplatelet therapy
**ATLAS ACS 2–TIMI 51**	Rivaroxaban 2.5 mg bid or 5 mg bid vs. Placebo	15526	Double Blind Phase 3	100%	61.7	Aspirin + Thienopyridine	13 month	Rivaroxaban superior to placebo for the study primary efficacy endpoint	Death from cardiovascular causes, myocardial infarction, or stroke	Major non-CABG related bleeding (TIMI)	Rivaroxaban significantly reduced the primary efficacy endpoint
**GEMINI ACS 1**	Rivaroxaban 2.5 mg bid vs. Aspirin	3037	Double Blind Phase 2	100%	62.3	clopidogrel/ ticagrelor	13 month	Estimate the bleeding risk of rivaroxaban compared with aspirin on top of standard P2Y12 inhibitor therapy	Cardiovascular death, myocardial infarction, stroke, or definite stent thrombosis	Non-CABG clinically signicant bleeding (TIMI)	Similar bleeding rate between rivaroxaban and aspirin on top of P2Y12i.
**COMPASS**	Rivaroxaban 2.5 mg bid + Aspirin Rivaroxaban 5 mg bid vs. Aspirin	27395	Double Blind Phase 3	0%	68	N.A.	23 month	Rivaroxaban superior to aspirin for the study primary efficacy endpoint	Cardiovascular death, myocardial infarction and stroke	Major or minor bleeding (ISTH)	Rivaroxaban 2.5 mg bid + aspirin significantly reduced the primary efficacy endpoint compared to aspirin alone
**COMMANDER HF**	Rivaroxaban 2.5 mg bid vs. Placebo	5022	Double Blind Phase 3	0%	66.4	N.A.	21 month	Rivaroxaban superior to placebo for the study primary efficacy endpoint	Death from any cause, myocardial infarction, or stroke	Fatal bleeding or bleeding into a critical space with a potential for permanent disability	No difference for the primary efficacy endpoint neither for the safety endpoint between rivaroxaban and placebo.
**APPRAISE**	Apixaban 2.5 mg bid or 10 mg od 10 mg bid 20 mg od vs. Placebo	1715	Double Blind Phase 2	100%	60.8	Aspirin + Clopidogrel	6 month	Explore the rate of bleeding with dose escalating Apixaban triple/dual therapy	Cardiovascular death, myocardial infarction, severe recurrent ischemia, or ischemic stroke	Major or clinically relevant nonmajor bleeding (ISTH)	Dose-dependent increase in bleeding events with Apixaban on top of antiplatelet therapy
**APPRAISE J**	Apixaban 2.5 mg bid or 5 mg bid vs. Placebo	150	Double Blind Phase 2	100%	64.6	Aspirin + Clopidogrel	6 month	Explore the rate of bleeding with dose escalating Apixaban triple/dual therapy in a Japanese population	Deaths, nonfatal myocardial infarction, unstable angina and stroke	Major or clinically relevant nonmajor bleeding (ISTH)	Dose-dependent increase in bleeding events with Apixaban on top of antiplatelet therapy
**APPRAISE II**	Apixaban 5 mg bid vs. Placebo	7392	Double Blind Phase 3	100%	67	Apirin + Clopidogrel	8 month	Apixaban superior to placebo for the study primary endpoint	Cardiovascular death, myocardial infarction, or ischemic stroke	Major bleeding (TIMI)	Apixaban increased the number of major bleeding without a reduction in ischemic events
**AFIRE**	Rivaroxaban 15 mg or 10 mg vs. Rivaroxaban + Antiplatelet therapy	2236	Open Label Phase 4	0%	74	Aspirin or Clopidogrel	24 month	Rivaroxaban monotherapy non-inferior for ischemia and superior for bleeding vs. Rivaroxaban + antiplatelet therapy	All-cause mortality, myocardial infarction, stroke, unstable angina requiring revascularization, or systemic embolism	Major bleeding (ISTH criteria)	Rivaroxaban monotherapy was non-inferior to the combination therapy for efficacy and superior for safety in patients with atrial fibrillation and stable coronary artery disease

Notes: ACS, acute coronary syndrome; bid, bis in die; CABG, coronary artery bypass grafting; F.U., follow up; GUSTO, Global Utilization Of Streptokinase And Tpa For Occluded Arteries; ISTH, International Society of Thrombosis and Hemostasis; MO, months; N.A., not available; od, once daily; TIMI, The Thrombolysis in Myocardial Infarction.

**Table 3:** Randomized controlled trials evaluating antithrombotic therapy type and duration in patients with non-valvular atrial fibrillation undergoing percutaneous coronary intervention.

	*N*	Randomization	P2Y12i Type	OAC Type	ACS	Age *	CHAD2Ds2 VASc	HAS BLEED	F.U.	Primary Endpoint	Results
**WOEST**	573	W-DT vs. W-TT	C (100%)	W	ACS 27%	70	1.5	N.A.	12 mo	Any bleeding (TIMI, GUSTO, BARC)	W-DT 19.4% vs. W-TT 44.4% HR 0.36 (95% CI 0.26–0.50) *p* < 0.0001
**ISAR TRIPLE**	614	W-TT 6 wk vs. W-TT 6 mo	C (100%)	W	ACS 32%	73	3.9	N.A.	9 mo	Death, MI, ST, stroke or major bleeding (TIMI)	6wkTT 9.8% vs. 6moTT 8.8% HR 1.14 (95% CI 0.68–1.91) *p* = 0.63
**PIONEER** **AF PCI**	2124	R-DT vs. W-TT vs. R-TT	C (94.3%)/ T (4.2%)/ *p* (1.3%)	W rivaroxaban 2,5 mg bid rivaroxaban 15 mg od	ACS 51.6%	70	n.a.	N.A.	12 mo	Major or minor bleeding (TIMI) or bleeding requiring medical attention	R-DT 16.8% vs. W-TT 26.7% HR 0.59 (CI 0.47–0.76) *p* < 0.001
**RE-DUAL PCI**	2725	D-DT110 or D-DT150 vs. W-TT	C (86%) T (12%)	W Dabigatran 110 mg bid Dabigatran 150 mg bid	ACS 50.5%	70	3.6	2.7	14 mo	Major or clinically relevant nonmajor bleeding event (ISTH)	D-DT110 15.4% vs. W-TT 26.9% HR 0.52 (95% CI 0.42–0.63) *p* < 0.001
											D-DT150 20.2% vs. W-TT 25.7% HR 0.72 (95% CI 0.58–0.88) *p* = 0.002
**AUGUSTUS**	4614	Factorial A vs. W DT vs. TT	C (92,6%) T (6,2%) *p* (1,1%)	W Apixaban 5 mg bid **	ACS 37.3%	70.7	4	2.9	12 mo	Major or clinically relevant nonmajor bleeding (ISTH)	A 10.5% vs. W 14.7% HR 0.69 (95% CI 0.58–0.81) *p* < 0.001
											DT 9% vs. TT 16.1% HR 1.89 (95% CI 1.59–2.24) *p* < 0.001
**ENTRUST AF-PCI**	1506	E-DT vs. W-TT	C (92%) T (8%) *p* (2%)	W Edoxaban 60 mg ***	ACS 52%	69	4	3	12 mo	Major or clinically relevant non-major bleeding (ISTH)	E-DT 17% vs. W-TT 20% HR 0.83 (0.65–1.05) *p* non-inferiority = 0.001 *p* superiority = 0.12

* Mean or median age in each trial is presented. ** 2.5 mg bid if dose reduction needed. *** 30 mg if dose reduction needed. N.A, Not Aviable; A, Apixaban; ACS, acute coronary syndrome; BARC, Bleeding Academic Research Consortium; bid, bis in die; C, clopidogrel; CI, confidence interval; D, Dabigatran; DT, dual therapy; E, Edoxaban; GUSTO, Global Utilization of Streptokinase and Tpa for Occluded arteries; HR, hazard ratio; ISTH, International Society of Thrombosis and Hemostasis; MI, myocardial infarction; mo, months; N.A., not available. OAC, oral anticoagulant; od, once daily; *p*, prasugrel; R, rivaroxaban; ST, stent thrombosis; T, ticagrelor; TIMI, The Thrombolysis in Myocardial Infarction; TT, triple therapy; W, warfarin; WK, weeks.

## Abbreviations

ACS	Acute coronary syndrome
AF	Atrial fibrillation
ASA	Aspirin
BARC	Bleeding Academic Research Consortium
BMS	Bare metal stent
CABG	Coronary artery bypass graft surgery
CAD	Coronary artery disease
CI	Confidence interval
DAPT	Dual antiplatelet therapy
DAT	Dual antithrombotic therapy
DES	Drug eluting stent
DOAC	Direct Oral anticoagulant
HR	Hazard ratio
ISTH	International Society on Thrombosis and Haemostasis
LAA	Left atrial appendage
LAAO	Left atrial appendage occlusion
MACCE	Major Adverse Cardiovascular and Cerebrovascular Events
MI	Myocardial infraction
NSTEMI	Non ST-segment Elevated Myocardial Infarction
OAC	Oral anticoagulant
P2Y12i	Receptor P2Y12 inhibitor
PAD	Peripheral arterial disease
PCI	Percutaneous coronary intervention
PFO	Patent foramen ovale
PTCA	Percutaneous transluminal coronary angioplasty
PV	Prosthetic Valve
RCT	Randomized controlled trial
SAPT	Single antiplatelet therapy
TAT	Triple antithrombotic therapy
TAVI	Transcatheter aortic valve implantation
TVMR	Transcatheter mitral valve repair
VKA	Vitamin k antagonist
